# Hypofractionated irradiation in elderly patients with breast cancer after breast conserving surgery and mastectomy : Analysis of 205 cases

**DOI:** 10.1186/s13014-015-0448-y

**Published:** 2015-08-04

**Authors:** Mélanie Doré, Bruno Cutuli, Patrice Cellier, Loïc Campion, Magali Le Blanc

**Affiliations:** Radiation Oncology Department, Institut de Cancérologie de l’Ouest, Nantes, France; Radiation Oncology Department, Institut de Cancérologie de Courlancy, Reims, France; Radiation Oncology Department, Institut de Cancérologie de l’Ouest, Angers, France

**Keywords:** Breast cancer, Hypofractionated radiotherapy, Elderly, Breast-conserving surgery, Mastectomy, Local recurrence, Toxicity, Nodal irradiation

## Abstract

**Background:**

Several randomized trials and meta-analyses confirmed a wide benefit of radiotherapy (RT), both after breast conserving surgery (BCS) and mastectomy. However, many elderly women don't receive RT. Hypofractionated (HF) RT allows « simplified » and more accessible treatments with equivalent results to classic RT in three large randomized trials. However, there are few available data on HF-RT for nodal irradiation, as well as for the boost.

**Methods:**

We evaluated patients treated for IBC by HF-RT between 2004 and 2012 in two regional cancer centres. We used an original scheme delivering 45 Gy in 15 fractions three times a week, both after BCS or mastectomy, with or without nodal irradiation. After BCS, a 9 Gy boost in 3 fractions was delivered. Local, regional and distant recurrences were assessed, as well as acute and late cutaneous, cardiac or pulmonary toxicities.

**Results:**

205 patients were analysed, 116 after BCS (57 %) and 89 after mastectomy (43 %). Median age was 81 years (range: 52-91); 44 % had axillary nodal involvement (pN+). The Nottingham Prognostic Index (NPI) scored 0, 1, 2 and 3 in 10 %, 27 %, 44 % and 19 % of the cases. A nodal HF-RT was delivered in 65 patients (32 %) and boost in 98 patients (84 % of BCS) by 9 Gy/3 fr scheme. Fifty (24 %) patients underwent chemotherapy and 156 (75 %) hormonal treatment.

With a 49-month median follow-up, 3/116 (2.6 %) patients and 4/89 (4.5 %) had local recurrence (LR) after BCS and mastectomy, respectively. The overall 5-year LR rate was 4.4 %. In univariate and multivariate analysis, LR risk factors were: high NPI (HR 5.46; *p* = 0.028), and triple negative tumour (HR 9.78; *p* = 0.006). Only 8 (4.5 %) patients had grade III skin toxicity; 29 (14 %) late fibrosis and 16 (8 %) telangiectasia. No pulmonary or cardiac toxicity was observed.

**Conclusion:**

Our HF-RT scheme (with or without nodal irradiation) confirms in elderly patients the data from randomized trials, both after BCS or mastectomy. Toxicity seems very acceptable but requires a longer follow-up. A larger evaluation is still ongoing in several other centres in France.

## Background

In Western countries, breast cancer (BC) is the most common female cancer, and occurs frequently in women older than 70 [1]. In two large national surveys performed in France (1155 patients) and Italy (3532 patients) in 2001–2002, BC rates in women older than 70 were 20.4 and 18.5 %, respectively [2, 3]. This rate reached almost 30 % in another Swiss study including 4820 patients treated from 2003 to 2005 [4].

The benefit of post-operative irradiation was widely confirmed by several studies, randomized trials [5, 6] and meta-analyses (EBCTCG) [7], both after breast conserving surgery and mastectomy, but many studies showed a clear undertreatment in older patients for various reasons (e.g. difficult access to radiotherapy centres, comorbidities). This fact induces higher local recurrence (LR) rates and increases long-term mortality [8, 9]. Thus, the International Society of Geriatric Oncology’s recent guidelines strongly recommend the use of post-operative RT in the same conditions as in a younger population, whenever possible [1]. In order to facilitate the access to radiotherapy centres and to simplify treatment modalities, several schemes of «hypofractionated» RT (HFRT) have been developed for 15–20 years, especially in UK and Canada [10–12]. Other French centres have been using «empirically» shortened schemes for many years [13–15]. However, due to a lack of data of the randomized trials on HFRT and mastectomy, HFRT and lymph node irradiation (LNI), and HFRT and chemotherapy, the use of a shortened scheme is not recommended yet in those situations.

## Methods

### Data collection

We assessed 205 postmenopausal patients treated by HFRT for a non-metastatic BC in two regional cancer centres (Nantes and Angers) between June 2004 and June 2012 both after breast conserving surgery (BCS) or mastectomy.

For each patient, a file detailed the following items: BC family history, age at menopause, hormone replacement therapy (HRT), comorbidities, type of surgery (lumpectomy/mastectomy), RT modalities (volumes/dose), chemotherapy and/or hormonal therapy.

The following histopathological features were also assessed: tumour size, axillary nodal involvement (ANI), histological subtype (ductal, lobular, mixed), SBR (Scarff, Bloom and Richardson) grading, excision quality, presence of vascular or lymphatic emboli, Her-2 oncoprotein over-expression and hormone receptor status (HR). The «Nottingham Prognostic Index» (NPI) score was calculated taking into account tumour size, ANI and SBR grading. The study was in accordance to the declaration of Helsinki in its latest version.

### Radiotherapy techniques and dose equivalence

HFRT was delivered 4–8 weeks after surgery or 4–6 weeks after chemotherapy. A 45 Gy total dose in 15 fractions (3 per week) was delivered to the whole breast (with or without a 9 Gy boost in 3 fractions) or chest wall, by two opposed tangential fields (4–10 MV photons). The boost was delivered by an anterior field (9–12 Mev electrons) or by two reduced tangential fields. In case of nodal irradiation (supraclavicular fossa (SCF), axilla or internal mammary chain (IMC)), the same dose was delivered (45 Gy/15 fr, 3 fr/week). SCF and axilla were treated by a 10 MV photon direct field (with or without a complementary posterior field for axilla according to dosimetry), and IMC by a direct field using a combination of photons and electrons. Assuming that α/δ = 3,5, the Biological Equivalent Dose (BED) of 45 Gy/15 fr scheme was 52 Gy using the iLQ version 1.0 Calculator (by SFjRO, French Society of Young Radiation Oncologist).

### Toxicity assessment and follow-up

During the treatment, a weekly consultation was performed by a radiation oncologist. The acute skin toxicity was assessed by the CTCAE scale (Common Terminology Criteria for Adverse Events). After treatment, the patients were assessed twice a year in order to evaluate the oncological outcome and possible toxicities (fibrosis, telangiectasias, rib fractures, heart or lung sequelea). An annual mammogram was performed as well as other exams in case of clinical symptoms.

### Statistics

The continuous variables were described by the median [extreme values] and/or the mean ± standard deviation. The qualitative variables were described by the distribution of their modalities. The evolutive variable (local recurrence-free survival) was defined as the period between the date of initial surgery and the date of local recurrence of the date of last news without local recurrence. It was calculated using the Kaplan-Meier method. The groups in question were compared using Student’s test (or the Mann-Whitney non-parametric test if necessary) for continuous variables and by Pearson’s Chi^2^ test (or Fisher’s exact test if necessary) for qualitative variables. The local recurrence-free survival curves were compared using the logrank test for the variables in classes and using the univariate Cox model for continuous variables. The prognostic variables at the univariate stage in addition to those with *p* < 0.15 were entered in the multivariate Cox’s semi-parametric regression model. The analyses were performed in bilateral formulation and the *p*-value of significance was set at 5 %. The software used was Stata 13.1 (StataCorp, College Station, Texas 77845 USA).

## Results

### Patients characteristics

Median age was 81 years (range: 52–91) with 94 % over 70 years; 45 patients (22 %) had BC family history; among all patients, only 18 (9 %) received HRT (median duration: 8 years). Among comorbidities, we observed heart failure, severe respiratory disease or severe neurologic or osteoarticular disease in 12, 5 and 32 % of the cases, respectively. BC was discovered clinically in 61 % of the cases; 56 % of BC were in the left side.

Histopathological characteristics of the entire population are described in Table 1. Histological median tumour size was 20 mm (3–70); 177 (86 %) were infiltrating ductal carcinomas (IDC), 14 (7 %) infiltrating lobular carcinomas (ILC), 4 mixed types and 10 other types; 90 patients (44 %) had ANI. Among 144 evaluated cases, 32 (22 %) had vascular emboli. Finally, 24 (12 %) tumours were triple-negative (ER-, PgR-, Her-2-) and 18 had Her2 over-expression (9 %).

**Table 1:** Histopathological features of the population

	Number of patients	Percent
pT		
1	85	41,5
2	87	42,5
3	15	7,5
4	11	5,5
not specified	7	3
pN		
0	115	56
1 (1–3 nodes)	63	31
2 (4–9 nodes)	20	10
3 (≥10 nodes)	7	3
HR positive (ER+ and /or PgR+)	172	84
HR negative	33	16
HER2 overexpression	18	9
Triple Negative tumor	24	12
SBR grading		
I	37	18
II	107	52
III	61	30
NPI class		
0	20	10
1	56	27
2	90	44
3	39	19

*HR* hormonal receptor, *ER* estrogen receptor, *PgR* progesterone receptor, *SBR* Scarff Bloom et Richardson grading, *NPI* Nottingham prognostic index

### Treatment modalities

Eighty-nine patients (43 %) underwent mastectomy and 116 (57 %) breast conserving surgery. For both groups, the median follow-ups were 53 and 47 months (*p* = 0,37), respectively. Table 2 summarizes radiotherapy modalities for both groups. Globally, 65 patients (32 %) underwent lymph node irradiation and 98 out of 116 (84 %) received boost after whole breast irradiation. Among 50 (24 %) patients undergoing chemotherapy (CT), 18 received a TC (Taxotere-Cyclophosphamide) protocol, 11 FEC 50 (5FU, Epirubicin, Cyclophosphamide) protocol, 9 EC 100-Taxotere and 12 others. Among 156 (75 %) patients undergoing hormonal treatment (HT), 150 received aromatase inhibitors and 6 Tamoxifen.

**Table 2:** Radiotherapy modalities among patients treated by mastectomy and breast conserving surgery (BCS)

	Mastectomy	BCS
*n* (%)	89 (43 %)	116 (57 %)
LNRT *n* = 65 (32 %)^a^		
Supraclavicular fossa (SCF)	40	24
Internal mammary chain (IMC)	16	6
axilla	5	3
Boost irradiation	4 (4.5 %)	98 (84 %)

*LNRT* lymphnodal irradiation with multiple fields possible

^a^multiple fields possible

### Local recurrences

After a 49-month median follow-up, seven local recurrences (LR) occurred: 3 out of 116 (2,6 %) patients treated by BCS and 4 out of 89 (4,5 %) treated by mastectomy. The overall 5-year LR rate was 4.4 %. One patient with LR after BCS had synchronous metastases and three patients with LR after mastectomy developed metastases in the subsequent 4 months. All the patients received chemotherapy (CT).

Table 3 details LR risk factors both in univariate and multivariate analysis. In univariate analysis, significant LR risk factors were high NPI (HR 5.46, *p* = 0.028), triple-negative tumour (HR 9.78, *p* = 0.006), with a trend for ANI (HR 6.97, *p* = 0.073). In multivariate analysis, only triple-negative tumours remained significant (*p* = 0.021) and high NPI was borderline (*p* = 0.058).

**Table 3:** Local reccurence risk factors

	Univariate analysis		Multivariate analysis	
Variable	HR	*p*	HR	*p*
	(95 % CI)		(95 % CI)	
Mastectomy	1.61 (0.36–7.19)	0.534	-	-
RT duration >42d.	0.50 (0.06–4.15)	0.521	-	-
ANI (pN+)	6.97 (0.83–58.2)	0.073	-	-
Delay between surgery and RT >42 d.	-	0.371	-	-
SBR III	2.95 (0.65–13.40)	0.161	-	-
Triple negative	9.78 (1.90–50.29)	0.006	7.19 (1.35–38.17)	0.021
Chemotherapy	6.81 (1.30–35.76)	0.023	-	-
Age	1.03 (0.90–1.19)	0.638	-	-
NPI (overall)	1.98 (1.08–3.65)	0.028	1.94 (0.98–3.86)	0.058
NPI 3 (vs 0-1-2)	5.46 (1.21–24.64)	0.027	-	-

*RT* radiotherapy, *ANI* axillary nodal involvement (pN+), *SBR* Scarff, Bloom and Richardson grading, *NPI*: Nottingham prognostic index, *d* days

### Regional and distant failures

Five nodal recurrences (NR) occurred : four in axilla and one in supraclavicular fossa. Two of these NR occurred after nodal irradiation. The overall 5-year NR rate was 3,6 %. Twenty-five patients developed metastasis with a 28-month median delay: 10 bone, 4 lung and 1 liver; 10 patients had multiple sites involved.

### Survival rates and death causes

The 3- and 5-year specific survival rates were 94 and 91.3 % respectively. Forty-two (20,5 %) patients died, including 15 by metastases, 24 by intercurrent disease and 3 of second cancer.

### Toxicities

Table 4 shows the early and late skin toxicity rates according to treatments.

**Table 4:** Skin toxicity

Toxicity			Breast		Chest wall	SCF^a^	IMC^b^
			Boost / No boost		wall		
			*n* = 98	*n* = 18	*n* = 89	*n* = 64	*n* = 22
Acute	Skin	Grade 1	54 (55 %)	9 (50 %)	70 (79 %)	29 (45 %)	14 (64 %)
		Grade 2	25 (26 %)	5 (28 %)	5 (6 %)	-	1 (5 %)
		Grade 3	6 (6 %)	2 (11 %)	-	1 (2 %)	-
Late	Skin	Fibrosis	24 (24 %)	3 (17 %)	3 (3 %)	-	-
		Telangiectasias	13 (13 %)	1 (6 %)	3 (3 %)	-	-
		hyperpigmentation	7 (7 %)	-	1 (1 %)	-	-

^a^Supraclavicular fossa

^b^internal mammary chain

Twenty-nine (14 %) patients had no acute skin toxicity; 133 (65 %), 35 (17 %) and 8 (4 %) had respectively grade I, II and III cutaneous toxicity (radio-epithelitis). Among 8 patients with grade III toxicity, only one underwent chemotherapy. Four patients with grade III toxicity required a 7–10 days stop during the treatment.

Thirty (14.6 %) patients had late fibrosis and 17 (8 %) telangiectasia. Telangiectasia was correlated to occurrence of grade II-III radio-epithelitis during the treatment (grade I: 5.6 % versus grade II-III: 19 %, Fisher’s test, *p* = 0.011). No cardiac or pulmonary toxicities were observed, neither were plexopathy or rib fractures.

## Discussion

BC in elderly women is an increasingly important issue, representing about 25 % of all BC [1–4]. Unfortunately, there are relatively few data on this population, because most of the trials exclude women over 70. However, several studies reported a clear “undertreatment”, both for locoregional and systemic treatment in elderly women, increasing the LR and death risks [9, 16–18]. Moreover, many physicians believe that BC in elderly is a “less aggressive” disease than in young women. This is true when compared to women under 40, but not with 40–69-year-old patients [19, 20]. Finally, distance from and/or rarity of radiotherapy centres and comorbidities are additional difficulties to optimize RT use in the elderly.

This is one of the main reasons of the development of “alternative/concentrated” RT schemes also called hypofractionated (HF) [21]. After many radiobiological analyses [22], several schemes were proposed, especially in UK and Canada. Three randomized trials confirmed equivalent results on local control and specific mortality between classical and HF-RT [10–12, 21] (Table 5).

**Table 5:** Description of the trials assessing hypofractionation (HF)

Reference	Type	Cohort (HF)	HF	Mastectomy	Boost	Lymph node RT	5-year LR
Whelan [12]	P.	622	42.5 Gy/16 fr	0 %	0 %	0 %	PFS 97.2 %
Owen [35]	P.	940	42.9 Gy/13 fr	0 %	75 %	21 %	7 %
			39 Gy/13 fr				9 %
Start A [11]	P.	1487	41.6 Gy/13 fr	15 %	61 %	13.2 %	3.2 %
			39 Gy/13 fr				4.6 %
Start B [10]	P.	1110	40 Gy/15 fr	8 %	43.8 %	7.4 %	2 %
Cutuli [2]	R. +CA	133	32.5 Gy/5 fr	0 %	55 %	48 %	3.7 %
Ortholan [14]	R.	150	32.5 Gy/5 fr	28.5 %	33,00 %	32 %	-
Kirova [15]	R. +CA	50	32.5 Gy/5 fr	0 %	0 %	-	-

*P* prospective randomized, *R* retrospective, *+ CA* with control arm, *Gr* gray, *fr* fractions, *PFS* progression-free survival, *LR* local recurrence

The Canadian trial HF-RT arm delivered 42,5 Gy in 16 fractions over 22 days [12], and included only women treated by BCS. The English START A Trial HF-RT arm delivered 41,6 Gy or 39 Gy in 13 fractions and 35 days, and included 85 % of BCS and 15 % of mastectomy [11].

In the subsequent START B Trial experimental arm, 40 Gy in 15 fractions and 21 days were delivered, 92 % after BCS [10]. These schemes are now validated by the French guidelines on infiltrating BC [23] for postmenopausal women with pT_1_T_2_N_0_ lesions and positive hormone receptors.

However, other schemes were used almost exclusively in elderly patients, often with many comorbidities. A single dose of 6,5 Gy per fraction each week (total: 5 or 6 fractions) was used at least in three reports [13–15, 24].

In France, another “empirical” approach was used in order to simplify treatment for elderly, with 42 or 45 Gy in 14 or 15 fractions over 4–5 weeks both after BCS or mastectomy. From a radiobiological point of view, assuming a 3.5 α/δ ratio for breast cancer, the biological equivalent dose (BED) of our scheme is approximately 52 Gy (classical fractionation) and 64 Gy if boost was applied. To our knowledge, our study is the first one evaluating this 45 Gy/15 fr/35 days scheme (+/− 9 Gy/3 fr. Boost), especially after mastectomy. It should be noted that in the START A and B trials 336 (15 %) and 177 patients (8 %) underwent mastectomy, but the data on nodal RT (supraclavicular fossa ± axilla, but never IMC) in these patients remain unclear, without technical details and no specific choice criteria. In a series from Thailand, 148 patients treated by mastectomy received HFRT, using an almost identical scheme to the Canadian Trial. With a 39-month follow-up, the local control rate was 86 %, without toxicity differences in comparison with 77 patients treated by a conventional scheme [25].

Despite a relatively short follow-up, our results seem to be consistent with the literature data, leading to an approximately 0,8 % LR rate a year [26, 27]. Acute cutaneous toxicity was acceptable with less than 5 % of grade 3 reaction. No long-term toxicity, particularly cardiac toxicity, was detected, whereas 56 % of the irradiations involved the left-hand side. Our results may be partly explained by a short hindsight, since cardiac toxicity is known to appear at a late stage. In the START trials, 0.8–1.4 % symptomatic pulmonary fibrosis, 1.3–1.5 % rib fractures and 2 % ischemic heart diseases were observed [10, 11]. In a study from Pakistan comparing three HFRT schemes, the cardiac toxicity was approximately 5 %, but the treatments were delivered only by Cobalt photons [28].

To date, there have been no recommendations relating to lymph node area irradiation in a hypofractionated schedule. Indeed, this was not performed in the Canadian trial or in the retrospective trial of Dragun [24], whereas lymph node irradiation was performed in 13.2 and 7.4 % of the patients in the START A and B trials respectively according to each centre’s policy. In our study, HFRT of the axilla, internal mammary chain and supraclavicular area were performed in 4, 11 and 31 % of the cases respectively. Nodal irradiation slightly increased the long-term cardiac death in several older studies, but the rates decreased widely with the use of modern RT techniques, such as clearly shown in the Danish trials widely using electrons to treat chest wall and more particularly IMC [29]. There was no significant influence of fraction dose [30]. In our series, no cardiac or pulmonary toxicities were observed, whereas 66 patients (32 %) underwent nodal irradiation. Unfortunately, we have no precise data on lymphoedema occurrence, but the few available literature data do not report any increased lymphoedema incidence among the patients treated by HFRT [31].

In our study, 84 % of the patients treated by BCS underwent a 9 Gy/3 fr boost, whereas there are no data on this modality in the literature, especially in women over 70. Due to the very low rate of LR, the impact of boost is not evaluable; as to skin toxicity, the rate is similar to those patients without boost, as well as for acute grade III radioepithelitis (6 vs 11 %) and late fibrosis (24 vs 17 %). This rate is quite similar to those observed (26 %) in boost arm in the EORTC Trial [32].

On the other hand, we found in this very old population only 37 % of deaths by breast cancer versus 63 % of deaths due to intercurrent disease, including second cancers.

This point is extremely important and has been confirmed by several other reports. In a Franco-Italian study including 927 patients over 70 treated by BCS + RT, the overall breast cancer death rate was 33 %, and such rate decreased according to age (36, 28 and 24 % in 70–75, 76–80 and >80 year groups), due to the influence of intercurrent diseases [13].

## Conclusions

Finally, our study confirms a good local control rate by HFRT in elderly women without severe toxicities similar to those observed in classical RT, even in case of nodal irradiation. These results confirm the results reported by others, including long-term data from meta-analysis [2, 10, 11, 14, 15, 33–35]. The impact of locoregional recurrence is clinically and psychologically important, even in elderly people [1], and except in case of very heavy comorbidities, an optimal treatment should be proposed in these women, including in case of LR risk factors boost after WBI. Our scheme is quite simple, similar to others used in randomized trials and seems very feasible and adaptable for many elderly patients.

A prospective survey on this RT modality is currently under evaluation in several other centres in France. However, HFRT must strictly comply with the optimal radiotherapy guidelines, both for breast and node irradiation, in order to avoid “hot spots” with a possible risk of long-term side effects, especially when associated to chemotherapy [36, 37].
